# Recombinant erythropoietin acutely decreases renal perfusion and decouples the renin‐angiotensin‐aldosterone system

**DOI:** 10.14814/phy2.13573

**Published:** 2018-03-04

**Authors:** Niels J. Aachmann‐Andersen, Soren J. Christensen, Kristian Lisbjerg, Peter Oturai, Pär I. Johansson, Niels‐Henrik Holstein‐Rathlou, Niels V. Olsen

**Affiliations:** ^1^ Department of Neuroscience and Pharmacology University of Copenhagen Copenhagen Denmark; ^2^ Department of Clinical Physiology, Nuclear Medicine and PET The Diagnostic Centre, Rigshospitalet Copenhagen Denmark; ^3^ Section for Transfusion Medicine Capital Region Blood Bank The Diagnostic Centre, Rigshospitalet Copenhagen Denmark; ^4^ Department of Surgery University of Texas Health Medical School Houston Texas; ^5^ Department of Biomedical Sciences University of Copenhagen Copenhagen Denmark; ^6^ Department of Neuroanaesthesia The Neuroscience Centre, Rigshospitalet Copenhagen Denmark

**Keywords:** Glomerular filtration rate, lithium clearance, recombinant erythropoietin, renal plasma flow, renin‐angiotensin‐aldosterone system

## Abstract

The effect of recombinant erythropoietin (rhEPO) on renal and systemic hemodynamics was evaluated in a randomized double‐blinded, cross‐over study. Sixteen healthy subjects were tested with placebo, or low‐dose rhEPO for 2 weeks, or high‐dose rhEPO for 3 days. Subjects refrained from excessive salt intake, according to instructions from a dietitian. Renal clearance studies were done for measurements of renal plasma flow, glomerular filtration rate (GFR) and the segmentel tubular handling of sodium and water (lithium clearance). rhEPO increased arterial blood pressure, total peripheral resistance, and renal vascular resistance, and decreased renal plasma flow in the high‐dose rhEPO intervention and tended to decrease GFR. In spite of the decrease in renal perfusion, rhEPO tended to decrease reabsorption of sodium and water in the proximal tubule and induced a prompt decrease in circulating levels of renin and aldosterone, independent of changes in red blood cell mass, blood volumes, and blood pressure. We also found changes in biomarkers showing evidence that rhEPO induced a prothrombotic state. Our results suggest that rhEPO causes a direct downregulation in proximal tubular reabsorption that seems to decouple the activity of the renin‐angiotensin‐aldosterone system from changes in renal hemodynamics. This may serve as a negative feed‐back mechanism on endogenous synthesis of EPO when circulating levels of EPO are high. These results demonstrates for the first time in humans a direct effect of rhEPO on renal hemodynamics and a decoupling of the renin‐angiotensin‐aldosterone system.

## Introduction

Recombinant human erythropoietin (rhEPO) and its analogs (erythropoiesis stimulating agents, ESAs) has been used for over 3 decades in the treatment of anemia. EPO is a glycoprotein mainly produced in the kidney that raises blood hemoglobin mass by anti‐apoptotic effects on erythroid progenitor cells in the bone marrow. The EPO receptor is also present in nonerythroid tissues including the brain, heart, kidney, and vascular endothelium, and the biologic actions of EPO extend beyond its effects on erythropoiesis. Numerous in vitro studies and experimental studies in animals have suggested that rhEPO has potential cytoprotective effects, as well as beneficial effects on inflammation and wound healing (Brines and Cerami 2005; Arcasoy 2008). The recognition of the pleiotropic properties of EPO has prompted the onset of clinical trials testing the potential of early treatment with high doses of rhEPO as a tissue‐protective agent.

Administration of rhEPO has been consistently shown to increase arterial blood pressure in both normal subjects and patients with chronic kidney disease, and presumably the increased cardiovascular risk is linked to the vasoconstrictive effect of rhEPO (Krapf and Hulter 2009; Lundby and Olsen 2011). The hypertensinogenic effects of rhEPO is independent of its erythropoietic effect (Krapf and Hulter 2009; Lundby and Olsen 2011) and we have previously shown that both acute short‐term, high‐dose rhEPO and prolonged low‐dose rhEPO augment systemic and cerebral vascular resistance well before increases in hematocrit (Lundby and Olsen 2011; Rasmussen et al. 2012). When assessed in isolated rat renal and mesenteric arterioles (Heidenreich et al. 1991) and human placental blood vessels (Resch et al. 2003) rhEPO has a direct vasoconstrictive effect that may involve nitric oxide, endothelin, and prostaglandin‐dependent mechanisms. Furthermore, rhEPO attenuates the increase in bleeding time induced by aspirin (Tang et al. 2007), a commonly used anticoagulant for patients at cardiovascular risk. rhEPO may induce a procoagulant state by stimulating thrombopoiesis, platelet activity and endothelial activation (Stohlawetz et al. 2000), and sudden death after stroke, myocardial infarction, or pulmonary embolism in elite athletes has been linked with illegitimate use of rhEPO (Tokish et al. 2004).

In addition to systemic vasoconstriction, the arterial hypertension may also arise from intrinsic renal effects of rhEPO. Acute administration of rhEPO in perfused isolated kidneys in a dose‐dependent manner decreased renal sodium excretion (Brier et al. 1993). We have previously reported that rhEPO promptly‚ and before any changes in blood volume and hematocrit take place‚ causes a downregulation of the renin‐aldosterone system (Olsen et al. 2011). Conceivably‚ activation of such a negative‐feedback system that serves to downregulate the endogenous renal synthesis of EPO in the presence of high levels of circulating rhEPO is expected to involve renal afferent arteriolar constriction. In rodents‚ administration of rhEPO acutely reduced renal cortical blood flow (Ishikawa et al. 1999; Coleman et al. 2006). Interestingly‚ plasma levels of endogenous EPO‚ even within the normal range‚ in patients with essential hypertension is inversely correlated with renal blood flow and positively correlated with arterial blood pressure and total peripheral resistance (Langenfeld et al. 1997; Schmieder et al. 1997).

Tubular segmental function can be evaluated with lithium clearance studies. Using renal lithium clearance as an index of proximal tubular fluid outflow (Thomsen 1984; Thomsen and Olesen 1984; Olsen 1998) it is possible to estimate the proximal reabsorption of sodium during rhEPO administration. The net result would be increased kidney oxygen tension causing a downregulation in endogenous EPO. This hypothesis fits well with the work of Lasne et al. who reported a decrease in renal excretion of endogenous EPO after administration of rhEPO (Lasne and de Ceaurriz 2000; Lasne et al. 2002). The use of the lithium clearance method in combination with renal clearance measurements of glomerular filtration rate (GFR) and renal clearances of sodium and water makes it feasible to elaborate on the effects of rhEPO on segmental tubular handling of sodium and water.

The link between administration of rhEPO and the renin–angiotensin–aldosterone system is interesting because the production of endogenous EPO, in part, may be regulated by this system. Several studies have demonstrated that the endogenous synthesis of EPO is stimulated by activation of an angiotensin II type 1 receptor‐dependent pathway (Freudenthaler et al. 1999, 2000; Gossmann et al. 2001). Other studies have shown good effects in treating polycythemia with angiotensin converting enzyme (ACE)‐inhibitors (Conlon et al. 1993; Gaston et al. 1993), perhaps by inhibiting sodium reabsorption leading to higher oxygen tension within the kidney and reduced EPO synthesis (Kristensen et al. 2009).

We speculate that rhEPO may have distinct effects on renal function besides the effect on the renin–angiotensin system. Treatment with rhEPO may result in suppression of endogenous EPO synthesis secondary to a decrease in intrarenal oxygen consumption. Conceivably‚ activation of such a negative‐feedback system that serves to downregulate the endogenous renal synthesis of EPO in the presence of high levels of circulating rhEPO is expected to involve renal afferent arteriolar constriction. rhEPO may directly downregulate the renin‐angiotensin system or perhaps the changes in the renin‐angiotensin system are secondary to changes in proximal tubular reabsorption. It is yet to be revealed how administration of rhEPO in humans is associated with changes in renal hemodynamics, GFR, segmental renal tubular function and sodium excretion.

## Material and Methods

In the present double‐blinded‚ placebo‐controlled study‚ we used classic renal clearance studies with renal vein catherization and constant infusions of ideal markers and urine collections to examine the effects in humans of both prolonged low‐dose and short‐term high‐dose rhEPO on renal plasma flow and GFR. In a cross‐over study, 16 healthy males, in random sequence, were given low‐dose Epoetin beta (5000 IU every second day for 2 weeks), high‐dose Epoetin beta (30,000 IU every day for 3 days), and placebo. We also used lithium clearance studies (Olsen et al. 2011) for evaluation of segmental tubular handling of sodium and water and performed sodium balance studies with the subjects at a low‐sodium diet advised by a dietitian. In addition‚ we assessed the hyperemic response to brachial artery occlusion by peripheral arterial tonometry (PAT) and measured venous blood concentrations of biomarkers reflecting endothelial and glycocalyx integrity and colloid osmotic pressure. Plasma volume, total hemoglobin mass and blood volume was estimated in single measurements at days 4, 11, and 25 during all 3 series by the use of the optimized CO‐rebreathing method (Schmidt and Prommer 2005).

### Subjects

Sixteen healthy male volunteers (age 25.4 [3.6] years, (mean [SD]), height 183.5 (6.6) cm, body weight 76.9 (7.3) kg and body mass index 22.9 (2.7) kg/m^2^) were included in the study. Before inclusion each subject underwent a medical examination and had to fulfill the following inclusion criterias: male gender, age between 20 and 40 years, nonsmoking, arterial systolic blood pressure below 140 mmHg and diastolic blood pressure below 90 mmHg, no actual medication, and body mass index <25. Exclusion criteria were participation in other studies, history of elite athletic performance, history of neoplastic diseases, polycythemia, epilepsy or allergy to rhEPO, and/or exposure to altitude (>1500 meters above sea level) within 3 months prior to the study. All subjects received 100% of the planned injections. One subject was excluded due to illness during the first wash‐out period. It was not characterized as an adverse effect by the GCP unit, and the subject's data are included in the statistical calculations.

### Experimental protocol

The effect of both low and high doses of rhEPO (Epoetin beta, NeoRecormon, Roche, Welwyn Garden City, UK) and placebo was evaluated in each subject by a randomized, double‐blinded, placebo‐controlled, cross‐over design with a 5 weeks wash‐out interval between the series:

1. Low‐dose rhEPO: 5000 IU (˜65 IU/kg) of subcutaneously administered Epoetin beta every second day for 2 weeks (days 1, 3, 5, 7, 9, 11, and 13; placebo on day 2).
2. High‐dose rhEPO: 30,000 IU (˜390 IU/kg) of subcutaneously administered Epoetin beta every day for 3 days (days 1, 2, and 3; placebo on days 5, 7, 9, 11, and 13).
3. Placebo (sodium chloride, isotonic 9 mg/mL, B.Braun, Melsungen, Germany) administered subcutaneously on days 1, 2, 3, 5, 7, 9, 11, and 13.

The primary endpoint in all series was changes in renal plasma flow (RPF) at days 4 and 25 and creatinine clearance and GFR at days 4, 11, and 25.

Randomization for the entire study (all 3 test series) was done before the beginning of the experiment. An independent investigator (NVO) generated a restricted randomization list and after inclusion each subject were given a number and a code, unique for subject and intervention. It was not possible for the subjects to visually distinguish between placebo or study medication. Randomization lists were sealed and not available for persons involved in endpoint registration. NVO did not participate in subject inclusion, study management, end‐point evaluation or data analysis. The study was approved by the Regional Committee on Biomedical Research Ethics, Committee B (protocol no. H‐2‐2011‐068) and the Danish Medicine Agency (EudraCT 2001‐005, 137‐39). The study was conducted according to the principles of Good Clinical Research (GCP), monitored by the GCP Unit at Copenhagen University Hospital (Bispebjerg Hospital) and registered on www.clinicaltrials.gov (NCT01584921). All subjects were informed verbally and in writing before the beginning of the study and gave written informed consent to participate. All experiments were done at the Copenhagen University Hospital (Rigshospitalet), Denmark.

All injections were administered subcutaneously between 08.00 and 11.00 am. The subjects did not receive any iron supplementation. Three days before each test day subjects refrained from excessive salt intake, according to written instructions from a dietitian. The subjects refrained from alcohol, caffeine‐containing drinks, and extensive exercise 24 h before each test day. Anticubital vein plasma samples were collected on day 4, 11, and 25, renal vein plasma samples on day 4 and 25. Blood samples were obtained after at least 60 min of rest in a sitting position using EDTA tubes and before administration of rhEPO. Immediately after centrifugation at 3000 *g* for 10 min, the plasma were stored at −80°C until analysis.

### Total EPO concentration

Plasma EPO concentrations were measured by means of the commercially available Quantikine^®^ IVD^®^ ELISA kit (R&D Systems Europe, Ltd., Abingdon, UK) according to the manufacturer's protocol which is based on the double‐antibody sandwich method. Assay results were measured spectrophotometrically at 450 nm using a microplate reader to determine the optical density. Duplicate readings were averaged for each standard, control, and specimen. The log of erythropoietin concentration was plotted versus the log of optical density for the standard curve. Concentrations are given in mIU/mL; samples with EPO concentrations above the range of the assay (2.5–200 mIU/mL) were diluted 10‐fold before a second analysis.

### Renal vein catheterization

The patient's right groin was prepped and drapped in the usual sterile fashion. Operators wore sterile gloves, caps, and mask. Large sterile drapes were placed over the insertion site, which was disinfected with chlorhexidine. All catheters were placed using the Seldinger technique. Right femoral vein puncture was achieved using ultrasound guidance. A percutaneous sheath introducer (Intro‐Flex, Edwards Lifesciences LLC, Irvine, CA, 6F [2.0 mm]) was placed in the vein and a Cordis (Johnson‐Johnson company) diagnostic catheter, 5F (1.65 mm), 65 cm, MPA 2 (open end, 2 sideholes) was inserted in the left renal vein. X‐ray verified catheter position along with a renal vein blood sample with a saturation above 85%. For acceptance of catheter position at the renal vein both parameters should be fulfilled. Renal vein catheterization was done on day 4 and 25.

### Urine collection

Subjects collected 24‐h urine samples before each test day during the study. The bladder was emptied just before start of each 24‐h collection period and time point for first and last voiding was noted. Urine was collected in preweighed containers and stored in a dark cool place. On arrival for the test day urine volume and density were determined and samples were divided into small eppendorf tubes frozen at −80°C until further analysis. During the 24‐h urine collections, we used Para Amino Benzoic Acid (PABA) tablets three times a day (240 mg/d), to verify completeness of the 24‐h urine collections (Bingham 2003). Acceptable completeness was a PABA recovery ≥90% (Bingham 2003). Statistical analyses were done only with complete collections (129 completed out of 139 possible urine collections).

### Renal Tc‐99 m‐DTPA and I‐131‐hippuran clearances

GFR was measured on day 4, 11, and 25 and RPF and ERPF were measured on day 4 and 25 by a constant infusion technique with urine collection and peripheral and renal vein plasma sampling. After an overnight fast, the clearance studies were started at 08.00 am by oral intake of bottled water to facilitate urine collections (500 mL/h without initial load). The water intake was maintained prior to and during tracer infusion to produce a state of water diuresis where the urine outflow approximately equaled the water intake. Except for briefly standing when voiding every 30 min, the subjects were confined to a resting sitting position.

Tc‐99 m‐diethylenetriamine pentaacetate (DTPA; Mallinckrodt Medical, Petten, the Netherlands), 4.8 MBq for GFR measurement, and I‐131‐hippurate (Polatom, Otwock, Poland), 0.3 MBq for RPF/ERPF measurements, were administered as bolus iv injections. At the same time a constant infusion of 500 mL isotonic glucose (162 mL/h) containing 6.7 MBq Tc‐99 m‐DTPA and 2 MBq I‐131‐hippurate was initiated. Resulting infusion rates were 0.037 MBq Tc‐99 m‐DTPA and 0.011 MBq I‐131‐hippurate per min. After a 60 min equilibration period and immediately before the investigation, the subjects emptied their bladder and the time was noted (*T* = 0 min). Thereafter, urine was collected for two 60 min clearance periods (U1 and U2) and plasma was sampled at the beginning and end of each period (*T* = 0, *T* = 60 and *T* = 120). Urine and plasma samples were counted on a gamma‐counter (Cobra II, Packard, Mriden, CT) using two energy windows and corrected for cross‐talk between the two isotopes. Using standard clearance equations renal clearances of the two tracers were calculated for each 60 min period and the averages of the two periods were used – Tc‐99 m‐DTPA clearance for GFR and I‐131‐hippurate for ERPF. RPF was calculated using the measured renal arterial‐venous plasma difference from sampled peripheral vein blood (≈arterial) and renal vein blood.

## Renal clearance measurement

Measurements of the renal clearance of lithium, used in this study as an index of proximal tubular fluid outflow (Thomsen 1984), were done simultaneously with the measurements of GFR, RPF, and ERPF on days 4, 11, and 25. Lithium carbonate (300 mg; 8.1 mmol) was given orally on the evening before each investigation Renal clearances of lithium (C_Li_) and sodium (C_Na_) were each calculated from U1 and U2 urinary excretion rates and representative values of plasma concentrations of lithium and sodium calculated as the mean from blood samples obtained at *T* = 0, *T* = 60, and *T* = 120 min.

### Colloid osmotic pressure

Plasma samples from the anticubital vein (*T* = 120 min) were analyzed in duplicates on colloid osmometer, Osmomat^®^ 050, Gonotec GmbH, GSG‐Hof Reuchlinstr. 10‐11, 10553 Berlin Germany.

### Analytical methods

^99m^Tc‐DTPA and ^131^I activity in plasma, urine, and standards was measured in a well gamma‐counter (Cobra‐II, Pachard Instrument Company, Meriden, CT). Plasma sodium was measured with a Technicon SMAC instrument, and urinary sodium was determined with a Technicon RA 1000 instrument (Tarrytown, NY). Plasma and urinary lithium were measured by atomic absorption spectrophotometry (model 403; Perking‐Elmer, Norwalk, CT).

### Calculations

Reabsorption and excretion rates of sodium and water were calculated based on the assumption that C_Li_ provides an accurate measurement of the rate of end‐proximal delivery of fluid and sodium (Thomsen 1984): Absolute proximal reabsorption rate (APR) = GFR − C_Li_; proximal fractional reabsorption (FPR) = 1 − (C_Li_/GFR); absolute distal reabsorption of sodium (ADR_Na_) = (C_Li_ − C_Na_) × P_Na_, where P_Na_ is plasma concentration of sodium; absolute distal reabsorption of water (ADR_H2O_) = C_Li_ − *V*, where *V* is urine flow rate; fractional distal reabsorption of sodium (FDR_Na_) = (C_Li_ − C_Na_)/C_Li_. Fractional excretions of sodium (FE_Na_), lithium (FE_Li_) and water (FE_H2O_) were calculated as C_Na_/GFR, C_Li_/GFR, and *V*/GFR, respectively. GFR of sodium and renal excretion rate of sodium were determined as GFR × P_Na_ and U_Na_ × *V*, respectively, where U_Na_ is the urinary concentration of sodium. Filtration fraction (FF) = GFR/RPF.

All calculations were done at both U1 and U2 period and a mean was used in the final calculations. Values are corrected for body surface area 1.73 m^2^.

### Finapres

The subjects rested sitting for at least 180 min after which HR (heart rate), MAP (mean arterial pressure), cardiac SV (stroke volume), TPR (total peripheral resistance) and thus Q were assessed noninvasively at heart level from the left third finger as an average over 90 sec using (Finometer^®^PRO, FMS Finapres Medical Systems BV, Hogehilweg 8, NL‐1101 CC Amsterdam ZO, the Netherlands) (Imholz et al. 1998; Buclin et al. 1999; Petersen et al. 2014).

### Plasma‐, blood volume and hemoglobin mass

Total hemoglobin mass was estimated in single measurements at days 4, 11, and 25 during all 3 series by the use of the optimized CO‐rebreathing method (Schmidt and Prommer 2005). Briefly, subjects rested for at least 180 min in order to stabilize blood volume (Ahlgrim et al. 2010). Three baseline capillary blood samples were collected from a preheated fingertip in 35 uL preheparinized tubes (Clinitubes; Radiometer, Brønshøj, Denmark). After baseline sampling the subjects inhaled a bolus of 1.2 mL per kg bw of chemically pure CO delivered via a 100‐mL plastic syringe (Omnifix, Braun, Melsungen, Germany) to a custom designed spirometer (Blood tec GbR, Bayreuth, Germany) creating a closed system. The system contained 3 L of pure oxygen, and was rebreathed for 2 min. Three blood samples were collected 7 min following the inhalation of CO using the same technique as during baseline sampling. Blood samples were immediately analyzed for percent carboxy hemoglobin (%COHb), [Hb], and hematocrit (Hct) on an ABL 800 blood gas analyzer (Radiometer, Brønshøj, Denmark). A CO analyzer (Draeger, Luebeck, Germany) was used to evaluate if a leak of the closed system occurred during the rebreathing period and measured any leftover CO in the spirometer and lung cavity following the rebreathing period.

Venous [Hb] and Hct were calculated using the results from the capillary blood and the following equations:

$$\left[\text{Hb}\right]={\left[\text{Hb}\right]}_{\mathrm{capillary}}\times 0.8787+1.2$$

$$\text{Hct}={\text{Hct}}_{\mathrm{capillary}}\times 0.8425+5.23$$

The difference in %COHb was used to calculate Hb_mass_ (Schmidt and Prommer 2005). Blood volume (BV), red cell volume (RCV) and plasma volume (PV) was calculated using the following equations:

$$\text{BV}={\text{Hb}}_{\mathrm{mass}}\left(g\right)\times 100/\left[\text{Hb}\right]\left(\text{g/dL}\right)$$

$$\begin{array}{cc} \text{RCV}= & \;{\text{Hb}}_{\mathrm{mass}}\left(g\right)\times 100/\text{mean corpuscular hemoglobin}\;\\ & \text{concentration}\left(\text{g/dL}\right) \end{array}$$

PV=BV−RCV

All measurements of Hb_mass_ and calculations of BV, RCV, and PV deviating more than two standard deviations from the mean were considered an error of measurement and excluded from all analysis. The coefficient of variance expressed as the percent typical error (%TE) was calculated using the following equation on the first two available measurements of Hb_mass_ during placebo treatment (e.g., day 4 and 11):

$$\begin{array}{cc} \%\text{TE}=\; & \text{standard deviation of the differences between}\;\\ & \text{measurements}/\sqrt{2} \end{array}$$

### Enzyme linked immunosorbent assay (ELISA) measurement

Soluble biomarkers of endothelial cell and glycocalyx activation and damage were measured in uniplicate by commercially available immunoassays in plasma/serum according to the manufactures recommendations: Histone‐complexed DNA fragments (hcDNA, Cell Death Detection ELISAPLUS, Roche, Hvidovre, Denmark; LLD not stated, relative quantification), soluble thrombomodulin (sTM, Nordic Biosite, Copenhagen, Denmark; LLD 0.38 ng/mL), syndecan‐1 (Diaclone SAS, Besancon, France; LLD 4.94 ng/mL), sVEcadherin (R&D Systems Europe; LLD 0.113 ng/mL).

### Digital pulse amplitude tonometry

We used an EndoPAT 2000 device (Itamar Medical Ltd., Caesarea, Israel) consisting of a fingertip plethysmograph (Faizi et al. 2009; Hamburg and Benjamin 2009). The device includes two fingerprobes, each placed on a fingertip on each hand. These are used for parallel measurements and are connected to a computer. The probe consists of a rigid external cap around an air‐filled chamber with a sensor. When the chamber is filled with air, a uniform pressure is provided which prevents the veno‐arteriolar vasoconstrictive reflex. The probe detects changes in volume in relation to the arterial pulsation and translates this to a peripheral arterial tone (PAT). A cuff was placed on the right arm always in which the measurement was performed. Measurements by the other probe left arm served as a control. Each measurement consisted of three phases: baseline, occlusion and reactive hyperemia. Baseline: The probe was set to inflate to 10 mmHg below diastolic pressure. Occlusion: The test arm was occluded to suprasystolic pressure for 5 min (Faizi et al. 2009). Reactive hyperemia: The subsequent increase in blood flow leads to a flow‐mediated dilatation, manifesting as reactive hyperemia, which was measured by the device as an increase in the pulse‐signal amplitude. The EndoPAT software calculates a post‐occlusion/pre‐occlusions‐ratio, the reactive hyperemia index (RHI). An RHI ≤1.67 is described as being abnormal by the manufacturer (http://www.itamar-medical.com/images/EndoPAT Multi Function USA.pdf).

### Statistical analysis

Statistical analyses were done by the use of the statistical SPSS software (IBM SPSS Statistics, version 20.0.0). Entries into the database of all data were verified by the GCP Unit at Copenhagen University Hospital. To assess the effect of rhEPO treatment a mixed general linear model for repeated measurements was used. (SPSS: Analyze>Mixed Model>Linear). Repeated measures and pairwise comparisons versus baseline were corrected for multiple comparisons (Bonferroni). Results are expressed as means (CI 95%). All calculations regarding values on day 4, 11, and 25 were done comparing each treatment and day against the mean of placebo values on day 4, 11, and 25. No significant carry‐over or period effect was found for any of the endpoints. The assumption of normally distributed residuals was evaluated by visual inspection of residual histograms. If necessary, data were log_10_ transformed before analysis. Two‐sided significance tests were used with a significance level of 5%.

## Results

### Comparable erythropoietic effects of low‐dose and high‐dose rhEPO

**Figure 1:**
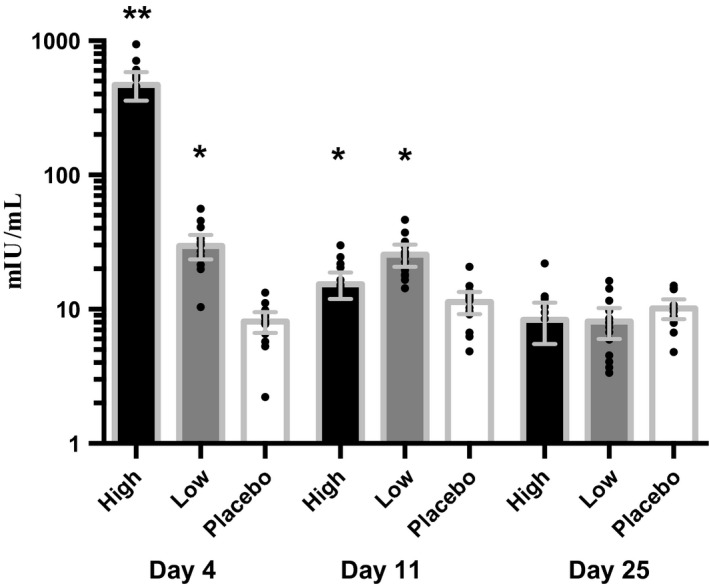
Total plasma EPO concentration (mIU/mL). *N* = 15. Log_10_ scale. Values are means [95% CI]. Data were log_10_ transformed before analysis. *P <* *0.004; **P <* *0.00001 compared with placebo. Data previously published by Aachmann‐Andersen et al., PLos ONE, 2014 vol. 9 (Bunke et al. 1994) pp. e110903. Absolute values are found in Table 1.

As expected both low‐ and high‐dose rhEPO administration increased total plasma EPO concentration throughout the treatment period but at day 25 the EPO concentration had returned to normal with a slight undershoot for both interventions (Fig. 1, Table 1). Both doses caused small increases in reticulocyte counts and hematocrit and a decrease in plasma levels of ferritin and transferrin saturation.

**Table 1:** Total plasma EPO concentration (mIU/mL). *N *=* *15

	Day 4	Day 11	Day 25
Placebo	8.1 (2.6)	11.3 (3.9)	10.1 (3.0)
Low‐dose EPO	29.7 (11.2)*	25.5 (8.6)*	8.1 (3.8)
High‐dose EPO	470.3 (202.0)*	15.4 (6.2)*	8.3 (4.9)

Values are means (SD). Data were log_10_ transformed before analysis. Data has been previously published in PLos ONE, 2014 vol. 9 (10) pp. e110903.

*P *<* *0.01 compared with placebo.

### Vasoconstrictive effect of rhEPO independent of erythropoiesis

**Figure 2:**
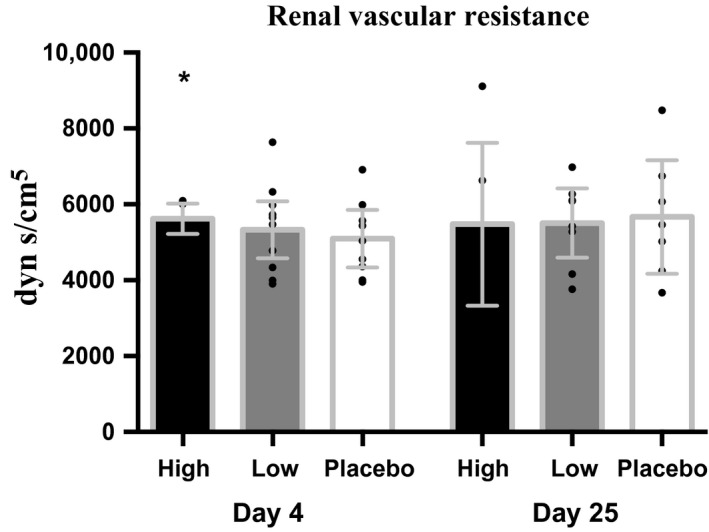
Renal vascular resistance (dyn s/cm^5^) after 4 and 25 days of either placebo, low‐dose rhEPO, or high‐dose rhEPO. *N *=* *16. Values are means [95% CI]. *P <* *0.05 compared with placebo. Absolute values are found in Table 6.

Studies with short‐term high‐dose rhEPO regimes have shown an increase in arterial blood pressure to the same level as low‐dose rhEPO treatment for 3 months (Rasmussen et al. 2010, 2012). In the present study, mean arterial pressure, diastolic pressure and total peripheral resistance increased with low‐dose rhEPO at day 11, whereas systolic blood pressure and stroke volume remained unchanged on all days. Renal vascular resistance increased with high‐dose rhEPO at day 4 (Fig. 2, Table 2). Plasma concentrations of nitrite and nitrate rose slightly at days 4 and 11 and returning to baseline at day 25 (Table 3). Ionized calcium remained unchanged compared with placebo (Table 4).

**Table 2:** Endothelial markers, RHI, and colloid osmotic pressure after 4, 11, and 25 days of either placebo, low‐dose rhEPO, or high‐dose rhEPO

	Day 4	Day 11	Day 25
Syndecan‐1 (ng/mL) *N *=* *7			
Placebo mean 44.7 [20.5–68.9]	47.8 [19.4–76.1]	45.0 [20.7–69.3]	41.3 [20.9–61.6]
Low‐dose EPO	39.8 [20.5–59.1]*	39.8 [16.8–62.9]*	40.2 [21.3–59.1]*
High‐dose EPO	41.2 [21.5–60.9]	38.3 [17.0–59.6]**	31.2 [16.6–45.9]**
Soluble trombomodulin (ng/mL) *N *=* *7			
Placebo mean 2.1 [1.2–2.9]	2.1 [1.3–2.9]	1.9 [1.1–2.7]	2.1 [0.8–3.4]
Low‐dose EPO^2^^,^ 3	2.2 [1.2–3.1]	2.1 [1.3–2.8]	1.5 [1.0–2.0]*
High‐dose EPO	1.8 [1.1–2.5]	1.9 [1.2–2.6]	2.2 [1.2–3.1]^4^
Vascular endothelial‐cadherin (ng/mL) *N *=* *7			
Placebo mean 41.9 [32.2–51.6]	35.9 [22.6–49.3]	52.5 [39.3–65.7]	37.1 [25.7–48.6]
Low‐dose EPO^1^^,^ 3	36.3 [23.9–48.7]	49.1 [36.7–61.4]	32.5 [24.0–41.0]
High‐dose EPO	36.7 [28.1–45.4]	45.3 [33.2–57.3]	39.4 [26.1–52.6]
Histone complexed DNA fragments (%) *N *=* *7			
Placebo mean 2.9 [1.1–4.6]	3.8 [1.2–6.3]	2.0 [0.5–3.4]	2.8 [1.2–4.5]
Low‐dose EPO^1^	2.0 [0.9–3.1]*	1.2 [0.7–1.7]**	1.5 [0.9–2.2]**
High‐dose EPO^1^^,^ 3	2.3 [1.2–3.5]	1.1 [1.0–1.1]*	2.2 [1.3–3.1]
Reactive hyperemia index (RHI)			
Placebo mean 2.3 [2.1–2.4]	2.2 [1.9–2.5]	2.6 [2.2–3.0]	2.1 [1.9–2.3]
Low‐dose EPO^1^	2.4 [2.0–2.7]	2.0 [1.8–2.2]	2.1 [1.9–2.4]
High‐dose EPO	2.3 [1.9–2.7]	2.1 [1.9–2.3]	2.2 [2.0–2.4]
Colloid osmotic pressure (mmHg)			
Placebo mean 26.0 [25.2–26.9]	26.3 [25.4–27.1]	25.9 [25.0–26.9]	25.5 [24.5–26.5]
Low‐dose EPO^3^	26.3 [25.4–27.2]	26.3 [25.0–27.5]	25.6 [24.3–26.9]
High‐dose EPO^2^^,^ 3	26.5 [25.8–27.3]	27.1 [25.9–28.2]*	25.5 [24.3–26.7]
Plasma volume (mL)			
Placebo mean 3653 [3394–3912]	3528 [3297–3760]	3639 [3235–4043]	3751 [3368–4133]
Low‐dose EPO	3355 [3043–3667]	3503 [3140–3867]	3626 [3296–3958]
High‐dose EPO	3412 [3147–3676]	3565 [3349–3779]	3711 [3456–3966]

*N *=* *16. Values are means [95% CI]. rhEPO, recombinant human erythropoietin.

*P <* *0.05; **P <* *0.01 compared with placebo.

^1,2,3^Difference between day 4–11, 4–25, and 11–25, respectively.

^4^Difference between high‐ and low‐dose rhEPO.

**Table 3:** Central hemodynamics after 4, 11, and 25 days of either placebo, low‐dose rhEPO, or high‐dose rhEPO

	Day 4	Day 11	Day 25
Systolic blood pressure (mmHg)			
Placebo mean 119.3 [114.4–124.2]	115.9 [108.5–123.2]	118.9 [109.9–127.9]	125.3 [116.8–133.8]
Low‐dose EPO	118.8 [111.4–126.1]	121.1 [111.4–130.9]	121.7 [115.1–128.3]
High‐dose EPO	121.6 [113.9–129.2]	115.3 [108.6–122.0]	120.2 [115.6–124.8]
Diastolic blood pressure (mmHg)			
Placebo mean 71.1 [69.2–73.1]	72.2 [69.2–75.2]	71.6 [66.5–76.7]	69.8 [67.6–72.0]
Low‐dose EPO^1^^,^ 3	72.7 [69.3–76.1]	77.6 [73.2–82.1]**	73.6 [71.7–75.6]
High‐dose EPO	70.6 [67.7–73.4]	72.6 [69.0–76.2]	69.8 [66.9–72.6]
Mean arterial pressure (mmHg)			
Placebo mean 88.0 [85.8–90.3]	88.0 [84.2–91.8]	88.4 [83.0–93.7]	88.4 [84.8–91.9]
Low‐dose EPO^1^	88.6 [84.7–92.5]	94.3 [89.1–99.4]**	90.5 [88.0–92.9]
High‐dose EPO	89.0 [85.7–92.3]	88.5 [84.4–92.6]^4^	87.6 [84.3–90.9]
Heart rate (beats/min)			
Placebo mean 57.6 [54.2–61.0]	58.2 [54.1–62.3]	54.6 [48.9–60.5]	55.5 [52.3–58.7]
Low‐dose EPO^3^	58.2 [53.3–63.1]	56.2 [51.5–60.9]	59.1 [54.4–63.9]
High‐dose EPO^1^^,^ 3	57.7 [52.8–62.6]	53.2 [49.1–57.4]*	57.0 [52.5–61.5]
Cardiac output (L/min)			
Placebo mean 5.2 [4.6–5.9]	4.9 [4.1–5.6]	5.2 [4.1–6.4]	5.5 [4.7–6.4]
Low‐dose EPO^3^	5.0 [4.3–5.7]	4.4 [3.4–5.4]	5.2 [4.7–5.7]
High‐dose EPO^1^^,^ 3	6.0 [5.1–6.9]^4^	4.7 [4.1–5.3]	5.7 [5.2–6.2]
Total peripheral resistance (dyn s/cm^5^)			
Placebo mean 1438 [1250–1625]	1556 [1263–1849]	1475 [1067–1884]	1312 [1126–1497]
Low‐dose EPO^3^	1532 [1284–1781]	1711 [1386–2035]*	1445 [1266–1624]
High‐dose EPO^1^^,^ 3	1266 [1084–1448]^4^	1570 [1373–1767]	1249 [1139–1359]
NOx (*μ*mol/L)			
Placebo mean 28.1 [22.2–34.1]	26.4 [18.6–34.1]	30.6 [22.9–38.3]	27.5 [22.1–32.9]
Low‐dose EPO	28.2 [20.8–35.6]	28.4 [23.3–33.4]	26.1 [22.4–29.7]
High‐dose EPO^3^	28.7 [21.1–36.4]	29.5 [23.3–35.8]	22.1 [19.0–25.1]

*N *=* *16. Values are means [95% CI]. rhEPO, recombinant human erythropoietin.

*P <* *0.05; **P <* *0.01 compared with placebo.

^1,2,3^Difference between day 4–11, 4–25, and 11–25, respectively.

^4^Difference between high‐ and low‐dose rhEPO.

**Table 4:** Hematological factors after 4, 11 and 25 days of either placebo, low‐dose rhEPO, or high‐dose rhEPO

	Day 4	Day 11	Day 25
Thrombocytes (×10^9^/L)			
Placebo mean 206.2 [190.6–221.9]	203.6 [180.3–226.9]	205.1 [186.8–223.3]	213.2 [198.1–228.3]
Low‐dose EPO^1^^,^ 3	200.4 [187.3–213.4]	233.1 [214.4–251.9]**	201.5 [178.5–224.5]*
High‐dose EPO^1^^,^ 3	212.4 [184.4–240.3]	248.9 [219.7–278.0]**	211.1 [192.9–229.2]
Ionized calcium (mmol/L) *N *=* *7			
Placebo mean 1.22 [1.20–1.24]	1.22 [1.19–1.26]	1.22 [1.21–1.23]	1.22 [1.20–1.25]
Low‐dose EPO^1^^,^ 2 ^,^ 3	1.24 [1.22–1.26]	1.20 [1.17–1.23]	1.22 [1.19–1.25]
High‐dose EPO^1^^,^ 3	1.22 [1.20–1.24]	1.19 [1.18–1.21]	1.21 [1.19–1.24]
Haptoglobin (g/L)			
Placebo mean 0.87 [0.71–1.02]	0.91 [0.72–1.10]	0.90 [0.69–1.11]	0.86 [0.65–1.07]
Low‐dose EPO	0.78 [0.60–0.95]	0.65 [0.51–0.79]*	0.73 [0.56–0.89]*
High‐dose EPO	0.72 [0.53–0.91]	0.73 [0.46–0.99]*	0.74 [0.52–0.96]
D‐dimer (mg FEU/L)			
Placebo mean 0.34 [0.30–0.37]	0.36 [0.27–0.45]	0.32 [0.29–0.35]	0.36 [0.28–0.43]
Low‐dose EPO^2^^,^ 3	0.39 [0.29–0.49]	0.43 [0.33–0.53]	0.55 [0.28–0.81]**
High‐dose EPO	0.35 [0.29–0.41]	0.33 [0.28–0.39]	0.41 [0.30–0.52]^4^
Activated partial thromboplastin time (sek) *N *=* *7			
Placebo mean 29.5 [27.8–31.3]	29.4 [27.7–31.2]	29.7 [27.5–32.0]	29.5 [27.2–31.8]
Low‐dose EPO	28.9 [27.4–30.3]	28.3 [27.5–29.2]	29.2 [28.2–30.2]
High‐dose EPO^3^	28.8 [28.2–29.4]	28.0 [27.3–28.7]	29.0 [27.7–30.3]
International normalized ratio			
Placebo mean 1.13 [1.08–1.19]	1.14 [1.07–1.22]	1.11 [1.08–1.15]	1.14 [1.07–1.22]
Low‐dose EPO^1^	1.14 [1.09–1.19]	1.20 [1.15–1.25]*	1.16 [1.08–1.23]
High‐dose EPO	1.18 [1.08–1.29]	1.16 [1.08–1.23]	1.14 [1.05–1.23]
Coagulation factor II, VII, and X (unit/L)			
Placebo mean 0.68 [0.62–0.75]	0.65 [0.58–0.72]	0.71 [0.66–0.76]	0.69 [0.60–0.77]
Low‐dose EPO	0.67 [0.60–0.74]^4^	0.62 [0.55–0.69]	0.66 [0.57–0.75]
High‐dose EPO^2^	0.61 [0.50–0.72]*	0.64 [0.57–0.72]	0.68 [0.59–0.77]

*N *=* *16. Values are means [95% CI]. rhEPO, recombinant human erythropoietin.

*P <* *0.05; **P <* *0.01 compared with placebo.

^1,2,3^Difference between day 4–11, 4–25, and 11–25, respectively.

^4^Difference between high‐ and low‐dose rhEPO.

Changes in glycocalyx and peripheral arterial tonometry (PAT) was used as an indicator of endothelial dysfunction. High‐dose rhEPO decreased at all days syndecan‐1 and histone complexed DNA fragments, whereas trombomodulin decreased at days 11 and 25 with low‐dose rhEPO (Table 2). D‐dimer and International Normalized Ratio (INR) remained unchanged on all days. Haptoglobin, activated partial tromboplastin time and coagulation factor II, VII, and X decreased on all days with high‐dose rhEPO at day 4 (Table 4) along with an unchanged reactive hyperemia index (RHI). rhEPO increased thrombocytes on day 11 (Table 2).

### rhEPO decreases renal perfusion and GFR

**Figure 3:**
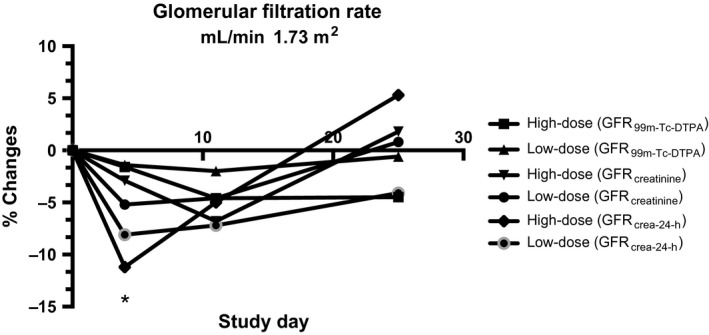
Glomerular filtration rate (relative change compared to baseline) after 4, 11, and 25 days of either placebo, low‐dose rhEPO, or high‐dose rhEPO. *N *=* *16. Values are means. *P <* *0.05 compared with placebo. GFR_T__c‐99 m‐_ _DTPA_ (Tc‐99 m‐diethylenetriamine pentaacetate, DTPA, infusion method); GFR_crea_ (Creatinine clearance during test day); GFR_crea‐24‐h_ (Creatinine clearance 24‐h urine collection). Absolute values are found in Table 5.

**Figure 4:**
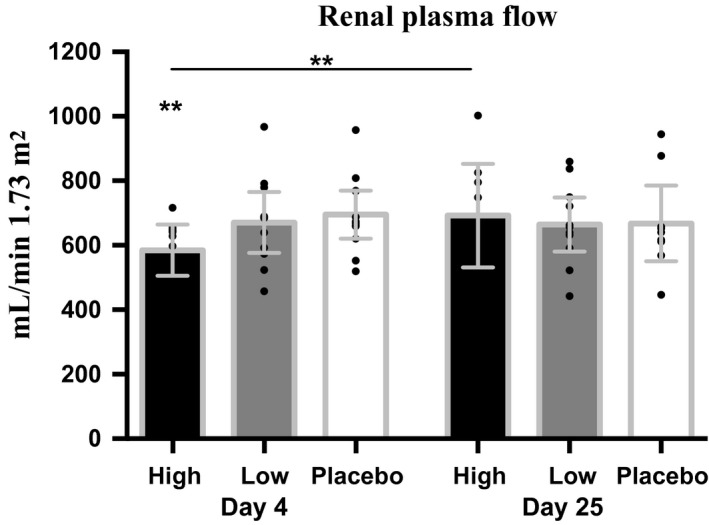
Renal plasma flow (mL/min 1.73 m^2^) after 4 and 25 days of either placebo, low‐dose rhEPO, or high‐dose rhEPO. *N *=* *16. Values are means [95% CI]. *P <* *0.05; **P <* *0.01 compared with placebo. Renal vein catherization and I‐131‐hippurate infusion technique. Absolute values are found in Table 6.

The GFR and renal plasma flow (RPF) were measured by classic clearance techniques with timed urine collections and the use of continous infusion of ideal markers (^99m^Tc‐DTPA for GFR and ^131^I‐hippurate for RPF). Furthermore, GFR was assessed by calculation of creatinine clearance (GFR_Crea_) for the same urine collection periods as for GFR_Tc‐99 m‐DTPA_, and by calculation of creatinine clearance for each 24‐h urine collection (GFR_crea‐24‐h_) preceeding the renal clearance studies. GFR_Tc‐99m‐DTPA_, GFR_Crea_, and GFR_crea‐24‐h_ decreased for both interventions at days 4 and 11 but only significantly for high‐dose rhEPO at day 4 measured by GFR_crea‐24‐h_; thereafter values gradually returned to placebo values at day 25 (Fig. 3, Table 5). To measure the overall rhEPO induced vasoconstrictive effect in the kidneys renal vein catheterization and infusion of I‐131‐hippurate were used to determine the renal plasma flow (RPF) (Hutchings et al. 2002). RPF decreased throughout the study period for both interventions returning to placebo values on day 25 (Fig. 4, Table 6). The changes in GFR and RPF resulted in an increase in filtration fraction for both interventions on all days.

**Table 5:** Renal clearance measurements after 4, 11, and 25 days of either placebo, low‐dose rhEPO, or high‐dose rhEPO

	Day 4	Day 11	Day 25
GFR_Tc‐99 m‐DTPA_ (mL min 1.73 m^2^)			
Placebo mean 105.8 [100.4–111.3]	103.8 [97.7–109.8]	108.7 [102.1–115.4]	106.1 [99.6–112.6]
Low‐dose EPO	104.3 [98.4–110.2]	103.7 [97.7–109.8]	105.2 [99.2–111.2]
High‐dose EPO	104.1 [98.7–109.4]	100.9 [92.8–109.0]	101.0 [95.8–106.0]
GFR_creatinin clearance_ (mL min 1.73 m^2^)			
Placebo mean 135.6 [124.7–146.6]	137.1 [126.6–147.7]	130.6 [117.0–144.4]	139.1 [126.1–152.1]
Low‐dose EPO	128.5 [116.3–140.6]	129.3 [116.0–142.5]	136.7 [126.5–146.9]
High‐dose EPO	131.6 [127.4–135.9]	126.4 [112.8–139.9]	138.0 [123.0–153.0]
Creatinine clearance during 24‐h urine collection (mL min 1.73 m^2^)			
Placebo mean 137.0 [123.0–150.9]	135.3 [121.2–149.5]	136.5 [118.8–154.1]	139.4 [122.2–156.6]
Low‐dose EPO	125.9 [116.6–135.3]	127.1 [116.6–137.6]	131.4 [119.4–143.4]
High‐dose EPO^2^^,^ 3	121.7 [110.0–133.4]*	130.2 [116.6–143.7]	144.3 [126.9–161.6]
Lithium clearance (mL min 1.73 m^2^)			
Placebo mean 23.3 [20.5–26.1]	23.0 [19.9–26.0]	24.3 [20.5–28.1]	23.8 [21.2–26.5]
Low‐dose EPO	23.5 [20.4–26.6]	23.1 [20.5–25.7]	24.1 [20.7–27.5]
High‐dose EPO	21.6 [19.4–23.7]	22.5 [17.8–27.1]	25.3 [19.9–30.7]
Absolute proximal reabsorption (mL min 1.73 m^2^) GFR_Tc‐99 m‐DTPA_ – C_Li_			
Placebo mean 82.2 [77.9–86.5]	81.7 [76.7–86.7]	83.1 [78.5–87.6]	82.0 [75.6–88.5]
Low‐dose EPO	81.2 [76.9–85.4]	80.1 [75.4–84.8]	80.7 [75.4–86.0]
High‐dose EPO^2^	82.9 [78.2–87.6]	78.2 [73.4–83.0]	76.6 [70.7–82.4]
Absolute proximal reabsorption (mL min 1.73 m^2^) GFR_creatinin clearance_ – C_Li_			
Placebo mean 111.7 [102.6–120.9]	114.7 [105.2–124.3]	106.4 [95.8–117.0]	114.2 [101.6–126.9]
Low‐dose EPO	105.0 [93.4–116.6]	106.2 [94.2–118.1]	112.0 [102.2–121.8]
High‐dose EPO	110.4 [105.6–115.3]	103.9 [94.2–113.6]	112.7 [99.0–126.4]
Sodium clearance (mL min 1.73 m^2^)			
Placebo mean 0.6127 [0.4768–0.7486]	0.6023 [0.4482–0.7564]	0.6805 [0.4635–0.8986]	0.5013 [0.3687–0.6340]
Low‐dose EPO	0.6164 [0.4518–0.7810]	0.6431 [0.4996–0.7865]	0.5041 [0.3771–0.6312]
High‐dose EPO	0.6000 [0.3970–0.8031]	0.6203 [0.3685–0.8720]	0.4788 [0.3124–0.6453]
Absolute distal reabsorption of sodium (mmol min 1.73 m^2^)			
Placebo mean 3.04 [2.66–3.42]	3.01 [2.60–3.43]	3.16 [2.64–3.67]	3.12 [2.76–3.48]
Low‐dose EPO	3.15 [2.71–3.60]	2.98 [2.64–3.33]	3.11 [2.67–3.56]
High‐dose EPO	2.79 [2.51–3.07]	3.03 [2.41–3.65]	3.29 [2.70–3.88]
Fractional distal reabsorption of sodium			
Placebo mean 0.973 [0.967–0.979]	0.972 [0.966–0.978]	0.971 [0.963–0.980]	0.980 [0.974–985]
Low‐dose EPO	0.971 [0.963–0.979]	0.974 [0.968–0.979]	0.978 [0.972–0.984]
High‐dose EPO	0.973 [0.965–0.982]	0.974 [0.965–0.983]	0.980 [0.974–0.985]
Absolute distal reabsorption of water (mL min 1.73 m^2^)			
Placebo mean 11.5 [9.8–13.3]	11.5 [9.3–13.6]	11.9 [9.4–14.5]	11.8 [10.2–13.3]
Low‐dose EPO^3^	12.9 [10.3–15.4]	11.6 [9.9–13.3]	13.6 [11.0–16.1]
High‐dose EPO^2^^,^ 3	11.1 [9.1–13.1]	11.6 [8.5–14.6]	14.5 [10.3–18.7]
Fractional proximal reabsorption			
Placebo mean 0.777 [0.759–0.796]	0.788[0.764–0.812]	0.766 [0.741–790]	0.772 [0.748–0.797]
Low‐dose EPO	0.784 [0.765–0.802]	0.773 [0.752–0.794]	0.772 [0.747–0.797]
High‐dose EPO	0.789 [0.769–0.809]	0.781 [0.752–0.809]	0.774 [0.738–0.809]
Fractional excretion of sodium			
Placebo mean 0.0057 [0.0044–0.0071]	0.0060 [0.0042–0.0077]	0.0063 [0.0041–0.0086]	0.0048 [0.0035–0.0061]
Low‐dose EPO^2^	0.0071 [0.0050–0.0092]	0.0064 [0.0050–0.0080]	0.0048 [0.0037–0.0060]
High‐dose EPO	0.0042 [0.0029–0.0055]^4^	0.0059 [0.0035–0.0082]	0.0042 [0.0030–0.0055]
Fractional excretion of water			
Placebo mean 0.1092 [0.1032–0.1152]	0.1057 [0.0971–0.1143]	0.1126 [0.1041–0.1211]	0.1096 [0.1009–0.1183]
Low‐dose EPO^3^	0.1054 [0.0993–0.1115]	0.1072 [0.0970–0.1173]	0.0998 [0.0917–0.1080]
High‐dose EPO	0.1051 [0.0947–1154]	0.1030 [0.0913–1148]	0.1011 [0.0920–0.1101]
Fractional excretion of lithium			
Placebo mean 0.2226 [0.2040–0.2412]	0.2120 [0.1878–0.2364]	0.2343 [0.2099–0.2587]	0.2279 [0.2033–0.2525]
Low‐dose EPO	0.2164 [0.1981–0.2348]	0.2268 [0.2057–0.2480]	0.2281 [0.2230–0.2533]
High‐dose EPO^2^	0.2110 [0.1908–0.2311]	0.2192 [0.1906–0.2478]	0.2358 [0.1970–0.2748]
Sodium excretion during test day (mmol day 1.73 m^2^)			
Placebo mean 118.1 [91.9–144.3]	116.7 [86.7–146.7]	131.0 [88.9–173.1]	96.2 [70.7–121.7]
Low‐dose EPO	118.4 [86.6–150.3]	122.9 [95.4–150.5]	96.3 [71.9–120.7]
High‐dose EPO	86.1 [61.4–110.8]	96.7 [55.0–138.3]	81.4 [55.8–107.1]
Filtration rate of sodium (mmol min)			
Placebo mean 14.17 [13.38–14.96]	13.92 [13.05–14.79]	14.58 [13.61–15.54]	14.17 [13.24–15.10]
Low‐dose EPO	14.33 [13.16–15.49]	13.77 [12.91–14.63]	13.98 [13.13–14.96]
High‐dose EPO	14.25 [13.22–15.28]	13.77 [12.85–14.69]	13.61 [13.09–14.12]
Urine flow rate during water diurese (mL min)			
Placebo mean 11.2 [10.1–12.3]	11.0 [9.8–12.2]	11.5 [10.1–12.9]	11.2 [10.1–12.3]
Low‐dose EPO	10.6 [9.6–11.6]	11.5 [10.0–13.1]	10.5 [9.4–11.6]
High‐dose EPO	10.4 [9.3–11.5]	11.0 [9.3–12.7]	11.0 [9.7–12.4]
Urine flow rate during 24‐h urine collection (mL min)			
Placebo mean 1.56 [1.30–1.82]	1.62 [1.25–1.98]	1.57 [1.25–1.89]	1.50 [1.21–1.80]
Low‐dose EPO	1.45 [1.15–1.76]	1.56 [1.24–1.89]	1.47 [1.20–1.74]
High‐dose EPO	1.49 [1.29–1.70]	1.56 [1.29–1.82]	1.65 [1.25–2.06]
Creatinine excretion during 24‐h urine collection (mmol day 1.73 m^2^)			
Placebo mean 15.39 [14.00–16.80]	15.68 [13.88–17.49]	15.20 [13.84–16.56]	15.40 [17.17]
Low‐dose EPO	14.90 [14.00–15.81]	15.31 [14.28–16.35]	15.34 [13.94–16.74]
High‐dose EPO^2^^,^ 3	14.01 [12.92–15.10]	14.28 [13.22–15.34]	16.04 [14.09–17.98]
Sodium excretion during 24‐h urine collection (mmol day 1.73 m^2^)			
Placebo mean 171.7 [145.2–198.3]	182.1 [140.6–223.6]	163.7 [131.8–195.6]	159.5 [104.9–214.1]
Low‐dose EPO	150.2 [124.2–176.2]	132.9 [111.9–153.9]*	116.8 [90.2–143.4]**
High‐dose EPO	126.5 [95.3–157.8]*	142.4 [110.4–174.4]	138.9 [109.5–168.4]
Tubular secretion of creatinine (mL min 1.73 m^2^)			
Placebo mean 28.1 [18.8–37.3]	28.0 [12.5–43.4]	27.3 [16.7–37.9]	28.9 [16.4–41.4]
Low‐dose EPO	21.1 [10.6–31.6]	22.9 [15.9–30.0]	29.1 [20.0–38.1]
High‐dose EPO^1^^,^ 2	12.6 [2.2–22.9]*	29.9 [18.7–41.0]	40.3 [17.9–62.6]

*N *=* *16. Values are means [95% CI]. rhEPO, recombinant human erythropoietin.

*P <* *0.05; **P <* *0.01 compared with placebo.

^1,2,3^Difference between day 4 and 11, 4, and 25, and 11 and 25, respectively.

^4^Difference between high‐ and low‐dose rhEPO.

**Table 6:** Renal hemodynamics after 4 and 25 days of either placebo, low‐dose rhEPO, or high‐dose rhEPO

	Day 4	Day 25
Renal plasma flow (mL min 1.73 m)		
Placebo mean 707.0 [627.0–787.1]	695.0 [620.0–770.0]	668.0 [550.6–785.3]
Low‐dose EPO	670.8 [576.0–765.6]	664.4 [580.8–748.1]
High‐dose EPO^1^	584.7 [505.2–664.2]**	692.4 [532.1–852.8]
Renal blood flow (mL min 1.73 m)		
Placebo mean 1227.3 [1068.4–1386.2]	1215.2 [1066.6–1363.8]	1126.5 [904.4–1348.7]
Low‐dose EPO	1170.8 [999.3–1342.2]	1179.3 [1031.2–1327.3]
High‐dose EPO^1^	997.1 [850.8–1143.4]*	1204.5 [918.3–1490.7]
Filtration fraction		
Placebo mean 0.148 [0.131–0.167]	0.149 [0.131–0.167]	0.157 [0.135–0.180]
Low‐dose EPO	0.163 [0.150–0.175]	0.160 [0.145–0.174]
High‐dose EPO	0.161 [0.144–0.179]*	0.159 [0.131–0.188]
Renal vascular resistance (dyn s/cm^5^)		
Placebo mean 5116 [4336–5897]	5093 [4335–5858]	5669 [4173–7165]
Low‐dose EPO	5328 [4579–6077]	5508 [4595–6422]
High‐dose EPO	5935 [5217–6017]*	5476 [3330–7623]
Renal fraction of cardiac output		
Placebo mean 26.5 [24.8–30.6]	28.8 [23.3–34.3]	22.7 [16.8–28.7]
Low‐dose EPO	28.0 [25.8–30.3]	28.0 [23.8–32.3]
High‐dose EPO	22.1 [16.3–27.9]* ^,^ 2	24.6 [18.0–31.2]

*N *=* *16. Values are means [95% CI]. rhEPO, recombinant human erythropoietin**.**

*P <* *0.05; **P <* *0.01 compared with placebo.

^1^Difference between day 4 and 25.

^2^Difference between high‐ and low‐dose rhEPO.

### rhEPO uncouples proximal tubular function and the renin‐angiotensin‐aldosterone system from renal hemodynamics

**Figure 5:**
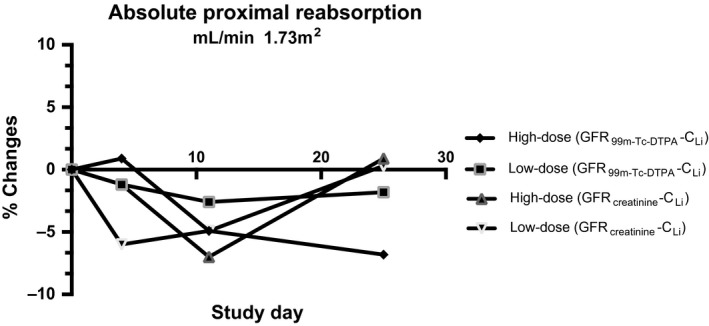
Absolute proximal reabsorption (relative change compared to baseline) after 4, 11, and 25 days of either placebo, low‐dose rhEPO, or high‐dose rhEPO. *N *=* *16. Values are means. *P <* *0.05; **P <* *0.01 compared with placebo. APR = GFR – C_L__i._ Absolute values are found in Table 5.

Lithium clearance was used as an index of proximal tubular fluid outflow (Thomsen 1984) simultaneously with the measurements of GFR and RPF. Lithium clearance remained unchanged on day 4 and 11 but increased nonsignificantly on day 25. These changes in GFR and C_Li_ resulted in a nonsignificant decrease in absolute proximal reabsorption of sodium and water (Fig. 5). Sodium excretion decreased throughout the study period for both high‐ and low‐dose rhEPO.

**Figure 6:**
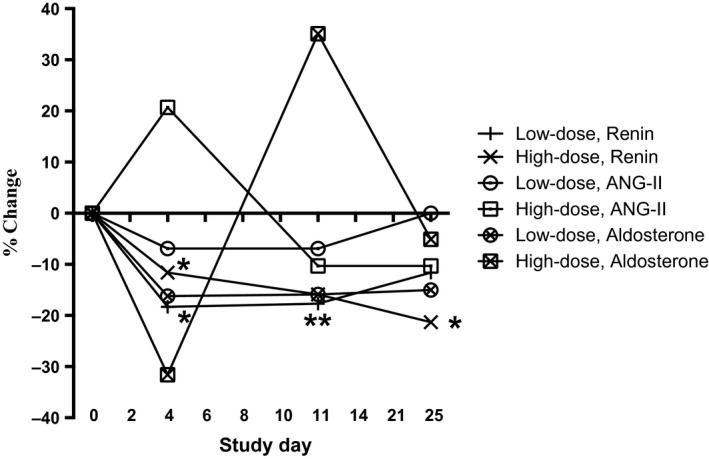
Renin‐Angiotensin‐II‐Aldosterone (relative change compared to baseline) after 4, 11, and 25 days of either placebo, low‐dose rhEPO, or high‐dose rhEPO. *N *=* *16. Values are means [95% CI]. *P <* *0.05; **P <* *0.01 compared with placebo. Absolute values are found in Table 7.

Plasma levels of renin, angiotensin‐II (ANG‐II), and aldosterone decreased promptly after administration of both doses of rhEPO and before rhEPO had induced any changes in hematocrit and hemoglobin (Table 2). Plasma renin concentration was decreased throughout the study to nadir level at high dose on day 4 and low dose at day 11. ANG‐II increased with high‐dose rhEPO at day 4 but thereafter decreased throughout the study. ANG‐II decreased nonsignificantly with low‐dose rhEPO at day 4 and 11 and returned to baseline values on day 25 (Fig. 6, Table 7). Aldosterone decreased nonsignificantly on day 4 and returned to baseline values after rhEPO treatment ended on day 25 with a slight overshoot.

**Table 7:** Renin, Angiotensin‐II, and aldosterone after 4, 11, and 25 days of either placebo, low‐dose rhEPO, or high‐dose rhEPO

	Day 4	Day 11	Day 25
Renin (pg/mL)			
Placebo mean 16.4 [6.4–26.5]	17.7 [4.7–30.7]	21.9 [2.9–40.8]	14.6 [4.3–24.8]
Low‐dose EPO	13.4 [6.5–20.2]*	13.5 [3.8–23.2]**	14.5 [3.7–25.3]*
High‐dose EPO	14.5 [2.6–26.5]*	13.8 [3.7–23.8]	12.9 [2.6–23.2]
Angiotensin II (pg/mL)			
Placebo mean 2.9 [2.3–3.5]	3.2 [1.7–4.7]	2.9 [2.3–3.4]	2.7 [2.4–3.1]
Low‐dose EPO	2.7 [1.9–3.4]	2.7 [2.2–3.1]	2.9 [2.5–3.3]
High‐dose EPO^1^^,^ 2	3.5 [2.9–4.1]	2.6 [1.7–3.6]	2.6 [2.0–3.2]
Aldosterone (pg/mL)			
Placebo mean 59.2 [0–138.0]	67.2 [0–178.3]	65.5 [0–173.7]	31.9 [0–50.6]
Low‐dose EPO	49.6 [0–132.4]	49.8 [0–103.2]	50.3 [11.5–89.0]
High‐dose EPO	40.5 [0–81.2]	80.0 [234.9]	56.2 [0–118.4]

*N *=* *7. Values are means [95% CI]. Statistics calculated on relative changes within each subject. rhEPO, recombinant human erythropoietin.

*P <* *0.05; **P <* *0.01 compared with placebo.

^1,2^Difference between day 4 and 11, 4 and 25, and 11 and 25, respectively.

Changes in hormones were accompanied by a nonsignificant decrease in plasma volume with nadir values for both interventions on day 4 (Table 2). Blood volume remained unchanged with a slight decrease on day 4. Red cell volume increased with maximum values on day 11. When rhEPO treatment ended, plasma volume, red cell volume, hematocrit and hemoglobin returned to baseline values. Colloid osmotic pressure remained unchanged at all days (Table 2).

## Discussion

This prospective, double‐blinded cross‐over study demonstrates for the first time a link between EPO and renal function in humans. Recently, we confirmed that rhEPO in normal subjects produces arterial hypertension (Rasmussen et al. 2012) and a reduction in plasma volume (Lundby et al. 2007; Olsen et al. 2011). The mechanism by which rhEPO raises blood pressure is yet to be fully understood, but may include inhibition of eNOS mediated NO synthesis (Wang and Vaziri 1999; Scalera et al. 2005), increased release of endothelin (Bode‐Böger et al. 1996) and degradation of endothelial function. Changes in the plasma volume is controlled via feed‐back mechanisms in the kidneys (Dunn and Donnelly 2007). Several studies have shown that administration of ANG‐II in humans stimulates EPO synthesis and conversely inhibitors of ACE and ANG‐II receptors decrease plasma concentration of endogenous EPO (Pratt et al. 1992; Gossmann et al. 2000). Our previous studies have shown that rhEPO suppress ANG‐II – in all this could indicate a negative feedback between these 2 hormones.

rhEPO decreased RPF and GFR nonsignificant in a dose‐dependent manner together with an increase in renal vascular resistance. The observed changes in renal hemodynamics resulted in increasing filtration fraction (GFR/RPF) at all days for both interventions, with highest values at day 4.

The clearance measurements revealed a decrease in proximal tubular reabsorption of sodium and a fall in GFR, after rhEPO administration. The combination of decreased GFR and unchanged C_Li_ results in a decreased fractional proximal reabsorption (1‐[C_Li_/GFR]) and absolute proximal tubular reabsorption of sodium (GFR‐C_Li_). This suggests that rhEPO reduces the reabsorption of tubular fluid in the proximal tubules thereby enhancing delivery of sodium, chloride, and water to the macula densa. An increased delivery of tubular fluid to the macula densa due to changes in proximal sodium reabsorption results in inverse changes in renin release (Briggs et al. 1990). Thus, the downregulation of renin, ANG‐II, and aldosterone may be secondary to a direct effect of rhEPO on the proximal tubules. The decrease in GFR may be a consequence of decreased proximal tubular reabsorption, which activates the tubulo‐glomerular feedback mechanism (Holstein‐Rathlou 1991) and triggers afferent vasoconstriction. However, a reduction in absolute proximal reabsorption should result in increased sodium urine excretion but opposite changes were measured. Therefore, compensatory sodium reabsorption in more distal part of the tubules, which is primarily load‐dependent (Subramanya and Ellison 2014), may be involved to compensate for the reduced sodium reabsorption in the proximal tubules.

Tubular reabsorption of sodium is the main oxygen consuming process in the kidney and around 70% of the filtered load is reabsorbed in the proximal tubule. By inhibiting proximal tubular reabsorption, which in turn results in rapid declines in GFR, rhEPO may directly reduce the major oxygen consuming factor in the kidney. By inhibiting sodium reabsorption tubular oxygen consumption is reduced and endogenous EPO production decreases due to a higher local oxygen tension. Our studies support these findings and we suggest that the renal effects of rhEPO may be part of a feedback system that serves to downregulate the endogenous renal synthesis of EPO in the presence of high levels of circulating EPO. In support of such a feedback system, evidence exists to indicate that prolonged administration of rhEPO results in suppression of urinary excretion of endogenous EPO (Lasne and de Ceaurriz 2000; Lasne et al. 2002). However, it is known that renal vasoconstriction as a result of Ca^2+^ influx causes a fall in renin secretion (Carlström et al. 2015). Together with the observed general vasoconstriction following rhEPO administration, we speculate that rhEPO could, in addition to the effect on the proximal tubule, also act directly on the juxtaglomerular cells, causing an increase in intracellular Ca^2+^ independent of the effect mediated via the tubule‐glomerular response. Also, the early rhEPO‐induced reduction in RAAS occurred before changes in plasma and blood volumes, indicating a direct effect of rhEPO on the proximal tubules and/or renin‐producing cells in the macula densa.

In support of a direct effect of rhEPO on the proximal tubules Eckardt et al. (1989) showed that blocking sodium reabsorption in the collecting duct, distal tubule, and thick ascending limb of the loop of Henle with amilorid, hydrochlorothiazide, and furosemide, respectively, did not change EPO production. But blocking sodium reabsorption in the proximal tubules with azetazolamide, however, led to a dose‐dependent decrease in EPO synthesis that was correlated with the increased natriuresis (Eckardt et al. 1989). This strongly indicates that the synthesis of EPO is linked to proximal tubular sodium reabsorption. These results suggest a link between rhEPO and renal tubular function. The understanding of rhEPO mechanisms of action on proximal tubular reabsorption is yet to be discovered, but may involve increased levels of endothelin‐1 (Bode‐Böger et al. 1996), which in low doses is known to attenuate sodium reabsorption in the proximal tubule (Clavell et al. 1995).

Normally a decrease in plasma volume/hypovolemia increases proximal tubular sodium reabsorption in order to compensate the lost volume. Changes in proximal reabsorption activates the tubuloglomerular feedback mechanism (TGF) which results in GFR changes. Decreased NaCl load at the macula densa normally leads to higher levels of renin, ANG‐II, and aldosterone. Its seems though that rhEPO induces a reduction in plasma volume and causes a hyporeninemic and hypoaldosteronism state paradoxically leading to a decrease in sodium excretion. We speculate that rhEPO may cause a downregulation of plasma volume and renal EPO isoform distribution (Aachmann‐Andersen et al. 2014) secondary to an inhibition of sodium reabsorption in the proximal tubules. The inhibition of renin must be a direct antagonistic effect of rhEPO as a reduction in plasma volume would tend to stimulate secretion. Yet rhEPO causes a reduction in proximal tubular reabsorption of sodium and seems to trigger the TGF and downregulate the RAAS. These opposing changes with decoupling of RAAS together with changes in the renal and systemic hemodynamics is not detected previously in humans.

We also found changes in biomarkers reflecting vascular endothelial glycocalyx damage. Endothelial glycocalyx is a complex carbohydrate‐rich layer of negatively charged anticoagulant membrane‐bound proteoglycans and glycoproteins covering the lumen of the vascular endothelium. Its main function is to protect and maintain the vascular barrier function and its integrity is important for vascular permeability (Reitsma et al. 2007). Degradation of endothelial glycocalyx has been demonstrated during global ischemia (Rehm et al. 2007) and early trauma‐induced coagulopathy resulting in increased values of syndecan‐1 (Johansson et al. 2012), trombomodulin (Ostrowski and Johansson 2012) and APTT causing hypocoagulopathy.

Syndecan‐1 has also been linked to cancer where it is used as an independent prognostic factor and seems to have a protumorigenic role (Gharbaran 2014). A damaged endothelial barrier changes the balance between intra‐ and extravascular volume, and increased permeability results in local edema, endothelial cell swelling, and necrosis (Johansson et al. 2011). These changes showing evidence that rhEPO results in impaired endothelial function, increased total peripheral and renal vascular resistance together with a systemic procoagulant state. EPO has been linked to hypertension, sudden death after stroke, myocardial infarction, and pulmonary embolism in elite athletes (Pope et al. 2014) which these findings support.

This study indicates that rhEPO increases arterial blood pressure and changes renal hemodynamics in a dose‐dependent manner resulting in decreased renal perfusion and GFR that was associated with an increase in total peripheral and renal vascular resistance.

Overall rhEPO causes hypertension and decreases RPF. There are two options that should be mentioned and they probably work together. First, the decline in RPF may be secondary to acute increase in renal vascular resistance and later general vasoconstriction. Second, the decrease in RPF may be due to the decrease in proximal tubular reabsorption and fall in renin and ANG‐II concentration. It is noteworthy that the general vasoconstriction occurs despite the decline in RAAS. Normally a decrease in RAAS would result in vasodilation and decrease in blood pressure (Ma et al. 2010) and several studies have reported that ACE inhibitors increase eNOS activity (Wassmann et al. 2002; Imanishi et al. 2008).

**Figure 7:**
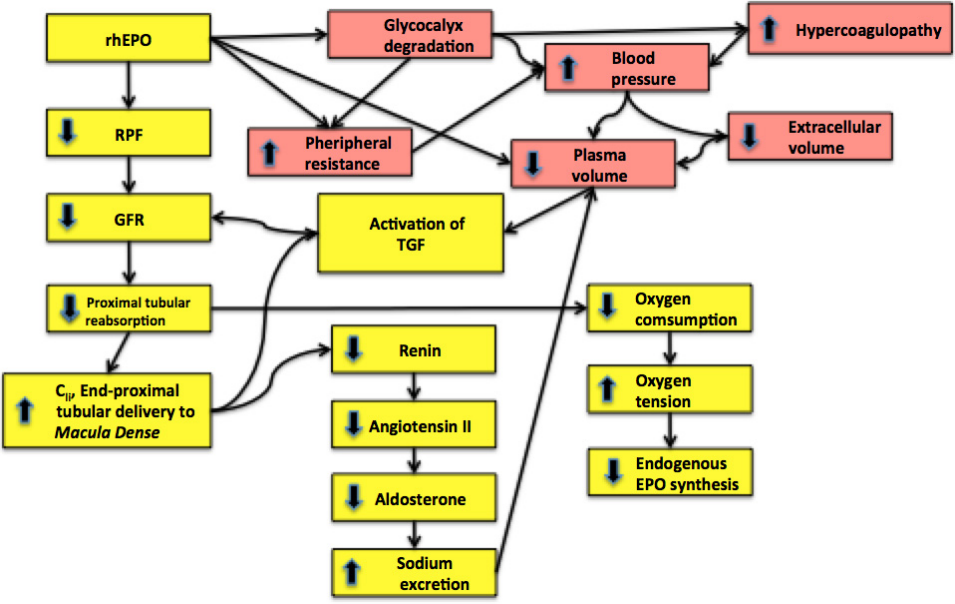
Suggestion on the mechanism involved in the changes in renal hemodynamics (yellow boxes) and systemic hemodynamics (red boxes).

Our results suggest that rhEPO may activate a pathway to downregulate the activity of the RAAS independent of changes in red blood cell mass, blood volumes and blood pressure, and to have a direct effect on renal hemodynamics (Fig. 7). These results demonstrate for the first time in humans a direct effect of rhEPO on renal hemodynamics and a decoupling of the renin‐angiotensin‐aldosterone system.

## Conflicts of Interest

The authors declare that they have no conflict of interest.
